# Bibliographical Notices

**Published:** 1853-04

**Authors:** 


### BIBLIOGRAPHICAL NOTICES.

*A System of Practical Surgery.* By WILLIAM FERGUSSON, F.R.S., Professor of Surgery in King's College, London; Surgeon to King's College Hospital; and Surgeon to H. R. H. Prince Albert. Fourth American, from the third and enlarged London edition. *With three hundred and ninety-three illustrations.* Philadelphia: Blanchard & Lea, 1853. 8vo. pp. 621, including index.

The fact that since its first appearance, ten years ago, the Practical Surgery of Prof. Fergusson has reached a third London and fourth American edition, affords abundant evidence of the great esteem in which it has been held on both sides of the Atlantic. The reputation of its author, as well as of the work itself, have been too long and too favorably familiar to our readers to require any detailed notice of the many new and strong claims upon their attention accumulated in the edition just presented to us. The marks of progress may be noted not only in the additional illustrations, of which there are over a hundred, but in the better arrangement as well as enlargement of several chapters, the discussion of recent improvements, and the consideration of many topics which were not alluded to or very slightly touched upon in previous editions. The nature and variety of these additions, however, must render it of course impracticable to dwell upon them here; and the best we can do for the book itself, as well as for our readers, is to recommend all who desire one of the most instructive manuals of practical surgery, to provide themselves at once with copies for their private reading. Meanwhile it may not be amiss to extract two passages from different portions of the book, which are interesting in themselves, and will serve to show the determination of the author to keep pace fully with the progress of the times. The first relates to the use of anæsthetics, and especially of chloroform, in operations. On this topic he remarks in conclusion :

"While the custom of rendering patients insensible to pain, during the performance of surgical operations, has rapidly gained an undoubted and sure position, there have been various questions and drawbacks regarding it, many of which are, even at the present day, of great practical importance. Several deaths have occurred, to all appearance directly from the anæsthetic agents, or the mode of their administration, and there has been much discussion on both these points. In the early application of ether, a complicated apparatus was used, but soon it was found that by holding over the patient's nostrils and mouth, a handkerchief, napkin or piece of sponge, moistened with a few drops of the anæsthetic, the desired effect was speedily produced, and to this day, perhaps the majority of those who use the agent are content with this mode of administering it. From the commencement it has been the custom of most surgeons in large operating practice, to have the agent administered by some one whose sole attention might be given to its physiological effects on the patient during the performance of the operation; and among those who have performed this most important duty, there are few so extensively experienced as Dr. Snow. This gentleman, who is justly deemed our highest authority on the subject of anæsthesia in surgical operations, is in the habit of using an apparatus, as he is of opinion that there is less danger and more certainty thereby than with a napkin. There are different views on this subject, however, which need not be discussed here, and it may be sufficient to say that in whatever way the agent is called into use, its powers are fraught with subtle and imminent danger, unless the greatest caution be taken. For my own part I think it of little consequence by what means the agent is administered, provided there be a fair proportion of atmospheric air admitted to the lungs at the same time, or that there be the facility of admitting it, when the influence of the chloroform, or whatever agent is employed, seems too powerful.

"At one time it was supposed that certain states of the constitution, certain conditions of the disease, or affections of certain organs, must preclude anæsthesia in surgical operations, but experience has shown that these fears have all been groundless. The age of a patient forms no objection to its use, for it may be given during the first few weeks of life, or at the latest imaginable date. Nor does it appear that positive disease of any organ forms an insuperable objection to its use. There are certain circumstances in the state of the constitution which will deter the surgeon from performing any important surgical operation, and in such instances the propriety of anæsthesia need never be discussed; but as a general answer to all questions on this score, I am disposed to say, that in whatever case a surgeon may consider an operation justifiable, the use of chloroform (this is named as the agent in most common use) is equally justifiable. If the operation be of minor importance, and of momentary duration, especially if the patient be courageous, and perhaps adverse to the practice, the surgeon need say little on the subject; but in all cases where much and protracted suffering is likely, chloroform should be recommended. It will not only save pain, but avert shock, and, provided the agent be properly administered, it will render the surgeon's duties more easy of accomplishment. There are few operations in which its use is not admissible and advantageous. In operations for cataract, especially by extraction, it is likely to prove injurious; as there is no certainty as to the movements of the eyeball and lids for some time after. I have used chloroform for removal of tonsils, in young persons, but the operation is difficult, and if the patient will keep steady otherwise, it had better not be used. In staphyloraphy the co-operation of the patient is so essential, that I deem chloroform inadmissible. It has been supposed that there is danger from blood trickling into the larynx and trachea in operations under chloroform. In one instance, in the early days of chloroform, I took some alarm on this score, in a case where blood was trickling from the posterior nares into the trachea and causing cough, but I feel now certain that there was no just cause of apprehension. I have since repeatedly removed both upper and lower jaw, and done many other operations involving considerable hemorrhage towards the throat, but have never seen any more inconvenience from such a cause than if chloroform had not been used. It has happened repeatedly in former times, that persons have died during the performance of operations. Mental emotions, shock, hemorrhage, have each and all had their influence; but, notwithstanding the various instances in which death has happened under chloroform, it may be doubted if sudden death, during operation, has occurred so frequently since the introduction of anæsthesia, as prior to that date. In my own experience I have twice seen patients under chloroform in great jeopardy—one of these had a protracted operation performed for resection of a joint, and possibly a similar state of prostration might have resulted without the use of this agent. In the other instance, tracheotomy was required for disease of the larynx, and under the chloroform, respiration became so slow and difficult, that it was feared the patient would die on the instant, before the operation could be executed. When the trachea was opened, he immediately breathed more freely, and speedily rallied from his alarming condition.

"I was certainly more impressed with the danger in this instance than in any other that I had seen; but Dr. Snow, who administered the chloroform, did not participate in my fears. The immediate effect of tracheotomy corroborates the view entertained by Ricord and others, that this proceeding may be resorted to with advantage when there seems danger from chloroform. When death has occurred seemingly from this agent, it has been immediate, and chloroform leaves the system so speedily, that if the patient has once become conscious, I doubt if death could ever be attributed to this cause. Chloroform has no influence whatever, either in retarding or accelerating the healing of a wound,

We have no statistical data on this subject, but altogether the impression on my mind is, that a patient is better off with chloroform both under and after an operation, than if he were without it. When an operation is contemplated under chloroform, almost the only precaution worthy of being taken is, that food should be avoided for several hours before, as chloroform on a full stomach is almost certain to induce sickness. So much has been written on the subject of chloroform since its introduction, that I deem it unnecessary to say much more regarding it at present, and I trust from what has been stated, that it will be understood that, with the few exceptions above made, and such others as might incidentally arise, the use of this agent is recommended as an invariable accompaniment of all surgical operations involving pain.

"The quantity of chloroform required for different patients is very variable. A few drops may suffice for some, while drachms may be needful for others, and generally the amount will be in proportion to the duration of the operation. Some persons take it kindly, even greedily, others make great objection and resistance to its use, both while conscious and unconscious, but all can be at last so thoroughly overpowered by its influence, that, compatible with respiration and circulation, the greatest imaginable quietness can be obtained. There is very little effect on the pulse in such cases; sometimes it is slightly excited at first, and if the dose be very potent, it may become slow and weak. There is greater effect on respiration. In some, in the early stages, the movements are rapid, and there seems an eagerness to fill the lungs with the vapor. In others, a tickling cough is excited. In many the full effect is induced with as slight a perceptible change as if sleep had suddenly come on, but in many others great irregularity in the respiratory movements ensues, and much cerebral excitement is induced. Rapid breathing may happen, as already mentioned, but the reverse may be the case, and the power of inspiration may suddenly seem to have ceased. For many seconds there may be no seeming attempt at respiration, but at last a deep breath is usually taken, which makes amends for the previous cessation. The cerebral excitement is evinced by crying, bawling, singing, laughing, incoherent talking, and with many there seems some prevailing idea which calls forth unusual and often great muscular action. Some strike forcibly with their fists, and with no seeming want of skill; others use their lower extremities, and others again make such violent general struggles as necessitate the greatest muscular exertion in those around to keep them steady. Often these violent struggles and the temporary cessation of respiration fall together, and to those not accustomed to such scenes, it appears as if immediate dissolution were impending; but in all such cases a little more of the vapor induces all that quietude which is characteristic of its full effect for the surgeon's purposes. There are various ways in which it may be decided that this period has arrived. Muscular relaxation is one of the best, but perhaps there is no test equal to that of touching the surface of the eyeball with the point of the finger. If there be any indication of feeling, the proper time has not arrived; if the contrary, then the surgeon may proceed. The agent must be administered during the operation as may seem necessary, and it is best to continue its influence until all risk of pain, at the time of operation or dressing, has passed. In most instances it is highly desirable that the patient should be placed in bed before awaking from his anæsthetic trance. In many cases much mental and bodily suffering may be avoided by administering the chloroform before the patient is moved from bed. In painful diseases of joints, such as of the knee, there is often great suffering in carrying the patient to the operating table, which may, however, be avoided by using chloroform before he is disturbed. Some patients become conscious in a few minutes after the surgeon has ceased his work, while others remain for a longer period as if in calm sleep. In certain instances headache and general uneasiness are complained of for hours or even days after chloroform; but in the majority of cases the effects are slightly if at all felt when consciousness returns." (pp. 33—35.)

The second extract contains his appreciation of the treatment of aneurism by compression.

"So far as our comparatively limited experience in the method by pressure, as followed by the Dublin surgeons, will enable us to form an estimate of its value, it seems in many respects, if not in all, preferable to that by deligation of the main artery; and there seems these great advantages in it, that if it does not act satisfactorily, the Hunterian operation may still be resorted to, with as much probability of success as ever, while by its application none of those formidable dangers are incurred, which are the well known consequences of the application of the ligature. The difficulties and immediate dangers of a cutting operation are avoided. It may seem strange to make use of such language in the present day, as applicable to ligature of the superficial femoral artery—for that I assume to be the vessel meant by the unfortunately vague term of 'femoral;' nevertheless when it is known that great difficulties have been experienced in such a proceeding, even by hospital surgeons and teachers of surgery, and that the accompanying great vein has been wounded by different operators, the facts cannot be overlooked. Mr. Syme states in the Edinburgh Monthly Journal of Medical Science for November, 1851, that he has tied the superficial femoral artery twenty times without a fatal issue, and with perfect success, and both he and his patients may be congratulated on such satisfactory results; but the Tables above referred to show no such average success; and when it is borne in mind that the attempt at cure by pressure does not preclude the resource of the ligature, it seems to me that with the ample evidence before us of the great success which has attended the modern practice by this means, the surgeon should undoubtedly give it a trial ere he resorts to the knife. Unquestionably the evil result of the Hunterian operation, in regard to ligature of the superficial femoral artery, has in many instances resulted from the defective or injurious style of operation, but the same may be said of certain examples where pressure has failed. Granting both of these statements to be correct, it cannot be overlooked that ligature of the superficial femoral artery, done to the perfection of human skill, has nevertheless been followed with the worst possible results. I have seen Mr. Syme perform the operation repeatedly with admirable skill and precision in all points, and the results have been all that could be desired; but I have seen many others, and among them I may name the late Mr. Liston, perform the same operation, with an equal amount of tact and judgment, yet the results have been very different. With pressure, surgery has still further resources, but with the ligature the fate of the case is, for a time, placed almost, if not quite, beyond human power; and doubtless the surgeons of Dublin who have resorted to this practice (many of whom stand among the highest of those who have graced the annals of the profession) have duly considered all these points." (p. 128.)

---

*Atlas of Pathological Histology.* By Dr. Gotleib Gluge, Professor of Physiology and Pathological Anatomy in the University of Bruxelles, &c. Translated from the German by Joseph Leidy, M. D., Pathologist to St. Joseph's Hospital, &c. *With three hundred and twenty figures, plain and colored, and twelve copper-plate engravings.* Philadelphia, Blanchard & Lea, 1853.

The student of pathological anatomy will not fail to recognize the name of Gluge, as one of the most industrious contributors to this department of medical science, and those familiar with his great work will also see, as an old friend, the present work originally appended to it.

Extending over only one hundred pages, it fulfils its title of *Atlas* in all particulars. The introduction occupying sixteen pages, gives us valuable tables of the magnitude and weight of the organs of man, in the normal and abnormal conditions.

A few introductory remarks on pathological histology open the division of the work devoted to this subject. It is divided into seven sections, each of which is clearly but succinctly expressed.

Section 1st treats of the development of the elements of tissues, and includes: 1, elements of tissues; 2, development of cells in the extended plasma of capillary vessels, in which the different modes of cell formation are treated of, and the parallelism between the pathological and physiological developments shown, together with the development of fibres.

Section II is entitled, "The elements of the tissues combined in perfect or imperfect tissues, and arranged according to the process of disease." It treats of the tissues and elements of the tissues in an imperfect condition of development; cytoblastema nucleoli, nuclei and cells. Of transition forms to perfect tissues. Of perfect tissues.

Section III treats of the formation of the blastema, the sources from whence the plastic substance is derived which forms the material for the development of the new tissues: these are, according to the author, nutrition, secretion and inflammation.

The different stages of inflammation are spoken of under the heads of congestion, hyperæmia, stasis, exudation and gangrene; each of which has a short section to itself; the formation, diagnosis, and chemical relation of pus, receiving a full share of notice.

Section IV is upon the histological metamorphosis of the blood, interior and exterior to the vessels.

Section V is devoted to the subject of pyæmia, and is illustrated by numerous observations.

Section VI is on gangrene, which it treats briefly.

Section VII contains observations on histology, illustrative of the various subjects treated in the preceding pages.

Next follows twelve well executed steel plates, containing three hundred and twenty figures in the best style of the art, with the explanation of each. Although there are many points where the author differs from standard authorities, yet, upon the whole, we are pleased with the book, and recommend it to all our readers who may be interested in this department. That it is entirely reliable, we cannot say, and by itself can do very little good or harm. No one will be satisfied with it alone, and a very slender acquaintance with other authorities will correct the errors of this. The translator has had many difficulties to encounter in rendering it into English, but he has performed his task creditably, and his few additions enhance the value of the work.

*Handbooks of Natural Philosophy and Astronomy.* By Dionysius Lardner, D. C. L., &c., &c. *Second Course. Heat, Magnetism, Common Electricity and Voltaic Electricity. Illustrated by upwards of two hundred engravings on wood.* pp. 451. Philadelphia, Blanchard & Lea, 1853.

The name of Dr. Lardner has long been known to the scientific world as one of the best of English writers on the various departments of the Physical Sciences. His treatises on the Steam Engine, and on other branches of physics, have for years been familiar in our schools and colleges as standard authorities.

The handbooks of Natural Philosophy and Astronomy, which are now in course of re-publication in this country, are designed by their author more especially for students of the different professions, for the artisan, and, in fine, for those who are already engaged in the active callings of life, but who desire to sustain and improve their knowledge of the general truths of physical science. They will be found admirably adapted to promote this end, by imparting, in a very clear style, a due amount of information without pursuing them through their mathematical details.

The study of the physical sciences form a part of every extended system of modern education. To the medical man, however, it is presented with peculiar claims to attention, since so many of the functions of the living body, and so many of the appliances which his art has suggested as remedial agents in disease, belong strictly to the domain of physics.

The present treatise is a most complete digest of all that has been developed in relation to the great forces of nature—Heat, Magnetism and Electricity. Their laws are elucidated in a manner both pleasing and familiar, and at the same time perfectly intelligible to the student. The illustrations are sufficiently numerous and appropriate; and altogether we can cordially recommend the work as well deserving the notice both of the practising physician and the student of medicine.

